# The use of single-point magneto-inertial measurement for gait and dynamic balance assessment of patients with Parkinson’s disease and Parkinsonisms: a systematic review with critical appraisal of clinical applications and quality of reporting

**DOI:** 10.3389/fneur.2026.1758979

**Published:** 2026-03-25

**Authors:** Lorenzo Cavazzuti, Benedetta Damiano, Andrea Merlo, Maria Chiara Bò, Sara Scaltriti, Francesco Cavallieri, Giulia Di Rauso, Giacomo Portaro, Giacomo Argenziano, Valentina Fioravanti, Isabella Campanini

**Affiliations:** 1 LAM - Motion Analysis Laboratory, Neuromotor and Rehabilitation Department, San Sebastiano Hospital, Azienda USL-IRCCS di Reggio Emilia, Reggio Emilia, Italy; 2 Merlo Bioengineering, Parma, Italy; 3 Neurology Unit, Neuromotor and Rehabilitation Department, Azienda USL-IRCCS di Reggio Emilia, Reggio Emilia, Italy; 4 Clinical and Experimental Medicine PhD Program, University of Modena and Reggio Emilia, Modena, Italy

**Keywords:** atypical parkinsonism, functional assessment, MIMU, Parkinson’s disease, wearable sensors

## Abstract

**Introduction:**

Gait and balance impairments are among the most disabling motor symptoms of Parkinson’s disease (PD) and parkinsonisms. Traditional clinical assessments are practical but often lack sensitivity to detect subtle functional changes and to monitor disease progression. Wearable magneto-inertial measurement units (mIMUs) offer an objective, portable, and low-cost alternative. Single-point mIMUs, positioned over the lower trunk, are particularly promising for clinical integration due to their simplicity. This systematic review critically evaluates how single-point mIMUs are used to assess functional mobility in PD and atypical parkinsonisms, and examines the quality and clinical transferability of current evidence.

**Methods:**

Following PRISMA 2020 guidelines, a systematic search was conducted in Medline, CINAHL, Embase, and Scopus (January 2025). Eligible studies included adults with PD or atypical parkinsonisms assessed with a single wearable sensor during straight-line walking and/or the Timed Up and Go test. Data extraction included clinical characteristics, assessment protocols, sensor specifications, and computed parameters. A customized critical appraisal tool evaluated five domains: methodology, clinical aspects, assessment protocol, technical aspects, and clinical transferability. Studies were classified as high-, medium-, or low-quality.

**Results:**

From 3,812 records, 41 studies met inclusion criteria, involving 395 participants (mean age 66 ± 5.9 years). Fourteen studies used the instrumented TUG, and 27 evaluated straight-line walking. Most studies were observational (90%). Only 12% achieved a high-quality rating, while 80% were medium-quality. “Technical aspects” was the best-rated domain, whereas “clinical aspects” and “assessment protocol” scored lowest, mainly due to incomplete reporting of sensor placement, acquisition conditions, computational methods, and clinical characterization of participants. Frequently reported outcomes included gait speed, step length, cadence, and TUG duration; however, methodological heterogeneity hindered cross-study comparability.

**Discussion:**

Single-point mIMUs can detect clinically relevant gait and mobility impairments and hold potential for monitoring disease progression, evaluating treatment effects, and distinguishing patient subgroups. Nevertheless, the overall quality of evidence is moderate, and insufficient methodological transparency currently limits clinical translation. Standardization of sensor placement, task instructions, data processing, and reporting—along with clearer clinical rationale and sample characterization—is needed to support reproducibility and facilitate adoption in routine practice.

**Systematic review registration:**

https://www.crd.york.ac.uk/PROSPERO/view/CRD420251025333.

## 1 Introduction

Parkinson’s disease (PD) is a complex neurodegenerative disorder that presents with a wide range of motor (e.g., tremors, bradykinesia, postural instability) and non-motor symptoms, including autonomic disorders and behavioral and cognitive deficits (1). Among the most debilitating motor impairments in PD are deficits in balance and gait, which markedly increase the risk of falls (2). Consequently, various clinical assessments have been developed to evaluate these symptoms. Commonly used tools include the Berg Balance Scale (3, 4), the Tinetti Gait and Balance Assessment (3), the Timed Up and Go (TUG) test (3, 5), the MiniBesTest (6) and the Postural Instability and Gait Disability (PIGD) score derived from the Unified Parkinson’s Disease Rating Scale (UPDRS) (3, 7). These instruments are not limited to fall-risk evaluation, but more broadly capture balance performance, mobility limitations, and disease-related motor features that may contribute to functional decline. In parallel, fall-specific and fear-of-falling measures (e.g., Falls Efficacy Scale–International) are often used to complement motor assessments by addressing the behavioral and psychological components associated with instability. These assessments are well-suited for clinical environments due to their low cost, minimal equipment requirements, and the immediacy with which results can be obtained and communicated to patients.

However, longitudinal studies have demonstrated that these tools possess limited sensitivity and specificity in identifying individuals at risk of falling within the PD population (3). Moreover, they may lack the resolution necessary to detect subtle changes in gait and balance among patients with mild to moderate disease severity (8–14).

In response to these limitations, research has increasingly focused on enhancing the objectivity and sensitivity of clinical assessments. Laboratory-based three-dimensional motion capture systems have traditionally been employed to analyze gait patterns in PD (15–20). Despite their precision, these systems are expensive, require expertise, and are impractical in smaller clinical settings due to their spatial and technical demands. In contrast, wearable sensor—including accelerometers and magneto-inertial measurement units (mIMUs)—have progressively emerged as valuable tools for the objective quantification of motor performance in Parkinson’s disease (21). Compared to traditional clinical scales, they offer several advantages, including portability, relatively low cost, ease of integration into routine assessments, and the ability to provide quantitative, high-resolution kinematic data in both laboratory and clinical environments.

Within this framework, recent studies have demonstrated that instrumenting standard clinical tests with wearable sensors can significantly enhance their sensitivity, enabling the detection of performance differences between individuals with PD and healthy controls (22–28).

A growing body of evidence supports the utility of wearable sensors in assessing balance and gait across a range of PD-related contexts: (i) comparisons between people with PD (pwPD) and healthy controls (22, 23, 29–34); (ii) discrimination between fallers and non-fallers with PD (35, 36); (iii) evaluation of different PD subtypes (26, 37–40); (iv) distinction between LRRK2 gene carriers and non-carriers (41); and (v) identification of individuals at high risk of developing PD (HRPD) (42, 43). Results indicate that wearable-derived metrics can effectively differentiate standing balance performance among HRPD subjects, PD patients, and healthy individuals (43). Logistic regression analyses further suggest that sensor-derived outcomes from tasks such as the instrumented Functional Reach Test can distinguish HRPD individuals from controls (42). Additionally, three-dimensional accelerometers placed on the head, trunk, or pelvis have revealed diminished gait rhythmicity in PD patients with a history of falls compared to non-fallers (35, 36). These findings collectively suggest that wearable sensors not only offer a valuable means of monitoring changes in postural control and gait, but also hold potential as screening tools for identifying PD-related motor risks, including fall susceptibility.

While the body of evidence supporting wearable sensor use in PD assessment is expanding, it is important to acknowledge that this field remains under development. The heterogeneity in methodologies across studies—including variations in sensor types, placements, and outcome measures—poses a challenge in identifying best practices (44, 45).

Among the various configurations, single-point mIMUs are increasingly attracting attention due to their practicality and potential for real-world use. Unlike multi-sensor systems, which require complex setups and are difficult to use outside controlled environments, single-point devices simplify data collection, enhance patient compliance, and allow continuous monitoring of gait and balance in daily life (46). While they do not capture the full spectrum of biomechanical parameters, validated single-point mIMUs have proven sufficiently accurate for clinically relevant spatio-temporal gait measures: these advantages suggest that single-sensor configurations may be critical for the wider clinical adoption of wearable technologies, highlighting the need for a focused review of their applications and limitations (46). This perspective guided our decision to focus on studies employing single-point inertial sensors. This approach aligns with similar reviews of other innovative methods (47, 48). While previous works have clearly highlighted the heterogeneity of outcomes used in gait and dynamic balance assessment research (49–54), none have offered practical guidance to establish a reference framework for improving the reporting of future studies.

This study aims to systematically review the literature on the use of single-point wearable sensors for assessing people with Parkinson’s disease and atypical parkinsonism, evaluate their clinical and technical aspects, and identify the gaps that hinder their translation into routine clinical practice. The findings will be used to provide recommendations aimed at improving the external validity and replicability of future studies.

## 2 Methods

This systematic review was conducted in accordance with the Preferred Reporting Items for Systematic Reviews and Meta-Analyses (PRISMA) 2020 guidelines (55).

To enhance the clarity, transparency, and reproducibility of this research, the protocol was prospectively registered in the International Prospective Register of Systematic Reviews (PROSPERO) on 3 April 2025 (ID CRD420251025333).

### 2.1 Research question and strategies

The leading question for this investigation was: “How can a single-point mIMU support the gait and dynamic balance assessment of patients with Parkinson’s disease and parkinsonisms?”

Two authors (LC and BD) developed comprehensive and systematic search strategies. The PICO framework was not applied because, for most of the studies included in this review, neither the Intervention nor the Comparison components could be clearly defined, as the aim was not to evaluate the effectiveness of a specific intervention on a particular outcome. Consequently, the search strings were formulated through an iterative process. The search was conducted in January 2025 across the following databases: MEDLINE, CINAHL, Embase, and Scopus. Additionally, a manual cross-reference search was performed by screening the reference lists of the included articles. No publication date limits were applied. The complete search strategies can be consulted as Supplementary material.

### 2.2 Eligibility criteria

The inclusion criteria were: (a) studies involving adult pwPD or other forms of atypical parkinsonism; (b) studies assessing pwPD using a single wearable sensor positioned on the lower back or sacral region included protocols such as straight-line gait and/or the “Timed Up & Go” test (56). These assessments are hereafter referred to as the “baseline conditions.” Additional conditions, such as cognitive or motor dual-tasks, were sometimes included alongside the baseline assessments when reported in the original studies; (c) primary peer-reviewed studies (e.g., RCT, clinical study, observational study); (d) full text available in English.

The exclusion criteria were: (a) studies not involving humans (i.e., modeling development using machine learning algorithm); (b) studies including only healthy individuals or patients with other pathologies; (c) acquisition setup based on more than one sensor; (d) sessions acquired only in an ecological context (e.g., real time at home monitoring); (e) studies published as abstracts and/or included in the proceedings of a conference.

A summary of the inclusion/exclusion criteria could be found in Table 1.

**Table 1:** Inclusion/exclusion criteria.

Inclusion criteria	Adults with Parkinson’s disease (pwPD) or atypical parkinsonism Assessment with a single wearable sensor placed on the lower back or sacral region during straight-line gait and/or Timed Up & Go (baseline conditions); additional dual-task conditions allowed if combined with baseline assessments Primary peer-reviewed studies (e.g., RCTs, clinical studies, observational studies) Full text available in English
Exclusion criteria	Non-human studies (e.g., modeling or machine learning development only); studies including only healthy individuals or patients with other pathologies Acquisition setup using more than one sensor; sessions conducted only in ecological/free-living contexts (e.g., real-time home monitoring) Conference abstracts and/or proceedings

We were interested in all outcome measures derived from gait and dynamic balance assessment acquired in the baseline conditions.

### 2.3 Study selection

After completing the database searches, all records were exported to EndNote20 (Clarivate Analytics, PA, USA) for duplicate removal. The remaining studies were then imported into the Rayyan online platform (57). Two independent reviewers (LC and BD) blindly screened the titles, abstracts, and full texts. Any discrepancies were addressed through a consensus meeting. When consensus could not be reached, a third reviewer (IC) was consulted to resolve the disagreement.

### 2.4 Data extraction and report

Two reviewers (BD and LC) independently extracted all relevant data. When the computation methods for specific parameters were unclear, a third author (AM)—a bioengineer with 20 years of experience in instrumental motion and posture analysis—was consulted for clarification. When additional information was required, the corresponding authors of the included studies were contacted by email; missing data were incorporated if a response was received within 1 month.

A custom table was created to summarize the following information: first author, year of publication, journal, study aims, sample size, demographic and clinical characteristics of participants, any treatments received, the protocol adopted for the gait and dynamic balance assessment (including testing conditions, number and duration of repetitions, and dosage of medications or brain stimulation), task conditions (e.g., execution speed, dual-task requirements), technical details of the instrumentation and data processing (e.g., filters applied), and all functional parameters reported under baseline conditions. Parameter values (means and standard deviations, medians, or ranges) were extracted from the text, tables, or derived from figures when necessary. The detailed data extraction table is available as Supplementary material.

### 2.5 Quality assessment

The three reviewers who completed the prior stages critically appraised the included studies. When questions emerged regarding the sample’s clinical characteristics, a neurologist with extensive expertise in pwPD was consulted (FC).

To standardize the quality assessment procedure, a tailored set of quality questions was designed inspired by a previous work by Merlo et al. (47), tested on a random sample of 30 studies, and refined progressively until its final version, reported in Table 2. The tool was developed iteratively by drawing on established methodological frameworks from prior reviews (48), ensuring coverage of methodology, clinical relevance, protocol details, technical specifications, and transferability—key aspects often underrepresented in standard instruments such as AMSTAR or QUADAS.

**Table 2:** Tailored critical appraisal for the assessment of the included studies.

Question	Domain	Question
Q1	Study methodology	Are the objectives of the study clearly stated? 0 = completely not stated 0.5 = authors described/introduced what they did 1 = the aim is clearly stated at the end of the introduction
Q2	Transferability to clinical practice	Are the objectives linked to daily clinical applications? 0 = no clinical application introduced 0.5 = clinical applications are not stated but can be derived from the introduction 1 = clinical applications are explicitly stated
Q3	Study methodology	Is the study design stated and the manuscript written accordingly? 0 = study design is not stated and the paper is not structured according to any appropriated checklist 0.5 = study design is not explicitly stated but the paper is structured according to the specific checklist 1 = study design is made clear in the manuscript and the paper is written accordingly
Q4	Clinical aspects	Are the characteristics of the sample sufficiently described (inclusion and diagnostic criteria, age, severity of the disease, etc.)? 0 = no disease-related information is provided to characterize the sample 0.5 = general inclusion criteria or sample characteristics are provided (diagnostic criteria only, or clinical scales only) 1 = sample is described according to diagnostic criteria, precise inclusion criteria, and clinical scales specific for the pathology (only for DBS study: stimulation parameters reported)
Q5	Study methodology	Are the sample characteristics adequate to address the aim of the study in terms of disease severity (e.g., homogeneity or stratification in descriptive and intervention studies, values distributed between minimum and maximum possible values in correlation studies)? NA = not applicable (for case studies or case series) 0 = no disease-related information is provided 0.5 = descriptive/intervention study includes a heterogeneous sample without stratification; correlation study includes a homogeneous sample but this represents only a subgroup of the population 1 = descriptive/intervention study includes a homogeneous sample or stratifies it; correlation study includes a heterogeneous sample representing the entire population
Q6	Study methodology	Is the included sample size justified by power computations or other methods, if applicable, or was an a-posteriori power analysis included? NA = not applicable (for case studies or case series) 0 = sample size was based on data availability and no a-posteriori power analysis was included 0.5 = sample size was based on data availability but a-posteriori power analysis was included 1 = sample size was a-priori designed based on power analysis computations
Q7	Clinical aspects	Are the characteristics of the rehabilitation treatment sufficiently described, when delivered (type of intervention, duration of each session, session weekly frequency, timeline and follow-ups….)? NA = not applicable (for observational studies) 0 = intervention is only cited 0.5 = intervention is described with few details (e.g., weekly frequency and duration) 1 = characteristics of the intervention are completely described to allow reproducibility
Q8	Technical aspects	Are the data acquisition and preprocessing parameters that may affect the signals clearly reported (e.g., sampling frequency, filters, sensors sensitivity\resolution)? 0 = no information is provided 0.5 = partial information is provided or can be retrieved from cited references 1 = parameters are entirely reported
Q9	Assessment protocol	Is the acquisition protocol sufficiently detailed to permit replication (path lengths, number of repetitions, sensor placement)? 0 = no information is provided 0.5 = partial information is provided or can be retrieved from cited references 1 = parameters are entirely reported
Q10	Assessment protocol	Are the task conditions sufficiently detailed to permit replication (walking pace, instructions to the patient, shoes\walking devices)? 0 = no information is provided 0.5 = partial information is provided or can be retrieved from cited references 1 = parameters are entirely reported
Q11	Assessment protocol	Are the testing conditions clear (ON/OFF drug condition, ON/OFF brain stimulation)? NA = not applicable for studies including patients diagnosed with parkinsonisms 0 = no testing information is reported 0.5 = testing conditions are cited 1 = information is reported in terms of dosage (LEDD or usual dosage), time of the day the drug was administered (washout clearly stated in case of OFF condition), hours from DBS starting
Q12	Transferability to clinical practice	Is the rationale for the selection of the assessment protocol (ITUG, IWALK, dual cognitive task) clearly stated? 0 = the rationale behind the choice of the protocol is not explained or cannot be derived across the manuscript 0.5 = the rationale behind the choice of the protocol can be derived across the manuscript 1 = the rationale behind the choice of the protocol is clearly stated
Q13	Transferability to clinical practice	Did the authors provide a rationale or discuss a-posteriori the choice of the parameters computed? 0 = the rationale behind the choice of the parameters is not explained or cannot be derived across the manuscript 0.5 = the rationale behind the choice of the parameters can be derived across the manuscript 1 = the rationale behind the choice of the parameters is clearly stated
Q14	Technical aspects	Was the computation of the parameters clearly described (definition, formulas, or reference to a paper with formulas)? 0 = definitions, formulas, or references for parameter computation are not reported 0.5 = some definitions, formulas, or references for parameter computation are reported 1 = definitions, formulas or references are provided for all parameters
Q15	Technical aspects	Are the units of measurement for each parameter clearly stated? 0 = units of measurements are never stated or are wrong 0.5 = units of measurements are stated only for few parameters or are not consistent across the manuscript 1 = units of measurements are clearly stated for each parameter
Q16	Technical aspects	Are the numerical data consistent with current literature? NA = not applicable when the parameter was used by fewer than five research groups^a^ 0 = data exceeding by 3 median absolute deviation (MAD) the overall median value 1 = data are consistent with the median and MAD derived from the total number of the studies
Q17	Study methodology	Are the limitations of the study discussed (control arm, sample size, power analysis, external validity, follow-up timeline, protocol feasibility)? 0 = limitations are not discussed 0.5 = only few limitations are reported with limited discussion 1 = limitations are properly addressed
Q18	Study methodology	Are the conclusions consistent with the aim of the study? 0 = conclusions do not align with the aim 0.5 = conclusions partially recall the aim of the study 1 = conclusions are in line with the declared aim
Q19	Transferability to clinical practice	Are the clinical utility and transferability of the assessment addressed and discussed in the study? 0 = no clinical utility or transferability are discussed 0.5 = clinical utility or transferability are no explicitly addressed but can be derived from the discussion 1 = authors made clear the clinical implications and transferability of their study

^a^Variables used by at least five research groups: Total TUG Duration (s), Sit-to-Stand Duration (s); Mid-Turning Phase Duration (s); Stand-To-Sit phase duration (s); Cadence (steps/min); Gait speed (m/s); Step Length (m); Step Time (s); Step time variability (%); Stride Duration (s); Stride Length (m); Stance (%); Swing (%); Double Support (%); Harmonic Ratio Antero-Posterior; Harmonic Ratio Medio-Lateral; Harmonic Ratio Vertical.

The questions, listed according to the traditional structure of the paper, explore five domains:

(1) Study methodology (Q1, Q3, Q5, Q6, Q17, Q18), including study design, research question, homogeneity or stratification according to disease severity, sample size or power analysis computation, and statistical analysis;
(2) Clinical aspects (Q4, Q7), including the description of the sample, and description of the intervention—if any;
(3) Assessment protocol (Q9, Q10, Q11), including information about gait and dynamic balance assessment procedures and their replicability;
(4) Technical aspects (Q8, Q14, Q15, Q16), including computational operations and engineering data management;
(5) Transferability to clinical practice (Q2, Q12, Q13, Q19), including the paper’s added value to the literature to improve clinical management of pwPD.

Each question was scored on a three-level basis: 1 for yes, 0.5 for limited details, and 0 for no (47, 48, 58, 59). For some items, a Not Applicable (NA) score was assigned. A total score was then calculated for each study to assess its overall quality. To avoid penalizing studies that received NA ratings, the score for each question—and consequently for each category—was expressed as a percentage of the maximum achievable score, considering only the items that were assessable.

Finally, studies were categorized into three groups: high-quality (total score exceeding 80%), medium-quality (total score ranging between 51 and 79%), and low-quality (total score below 50%) as in Samadi Kohnehshahri et al. (48). We also evaluated the quality of each category separately, based on the ratio of question scores at each level (low, medium, high) relative to the total number of questions associated with that category.

### 2.6 Differences in critical appraisal scores due to the journal subject area

We also examined whether studies published in strictly clinical journals differed in critical appraisal scores from those published in mixed journals (i.e., clinical and bioengineering, bioengineering only, or neurophysiology). Journals were classified as clinical or mixed based on their primary “subject area and category” listed on the Scimago Journal and Country Rank portal.^1^ For journals not indexed on Scimago or classified as “multidisciplinary” (e.g., PLOS ONE), classification was determined by reviewing the journal’s aims, the study content, and the authors’ affiliations. Critical appraisal analyses were subsequently performed separately for these two subgroups.

### 2.7 Differences in critical appraisal scores based on the examined functional task

We further investigated whether studies analyzing different functional tasks obtained different scores in the critical appraisal. Specifically, we compared studies focusing on the instrumented Timed Up and Go test (iTUG) and those assessing instrumented walking (iWALK). This comparison was performed to explore whether the nature and complexity of the examined motor task could influence the methodological quality of the studies. Indeed, iTUG and iWALK differ in task characteristics and experimental approaches: yet, in our opinion, these differences could influence study design and data analysis, potentially affecting critical appraisal scores.

## 3 Results

**Figure 1:**
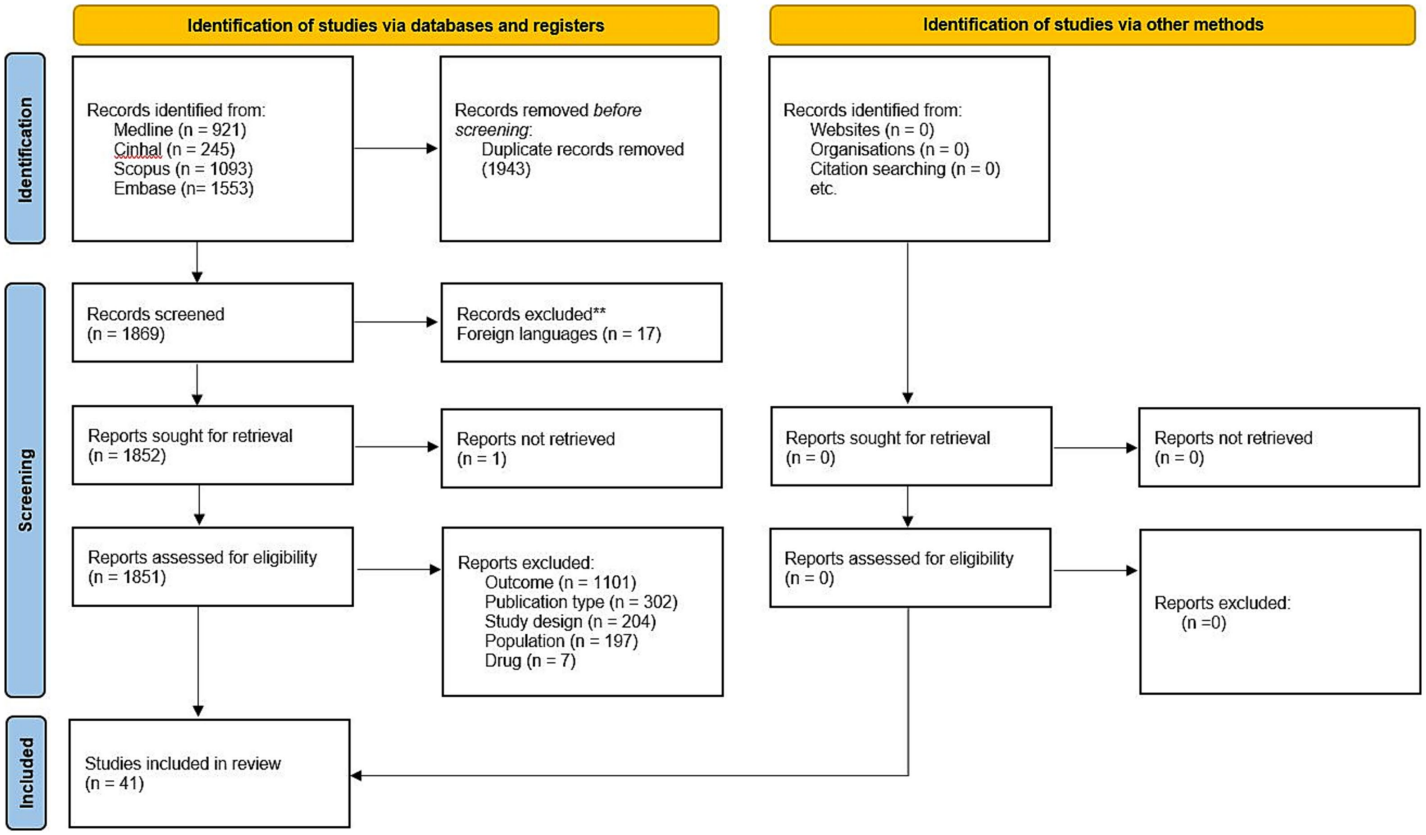
PRISMA flowchart of the literature search.

The initial search yielded 3,812 articles, which was reduced to 1,869 after duplicate removal. These records were screened based on titles and abstracts according to the eligibility criteria. The primary reasons for exclusion at this stage were the use of inappropriate outcomes or protocols (e.g., studies not including baseline conditions or employing more than one sensor) and unsuitable publication types (e.g., conference abstracts). 1,851 studies were assessed as eligible papers. No other studies were identified by hand searching. 41 studies were finally included in this review (see the PRISMA Flowchart in Figure 1) (23, 25–27, 30, 34, 41, 60–92).

### 3.1 Data extraction

Of the 41 studies, 40 focused on pwPD alone and one on pwPD and atypical parkinsonism (i.e., PSP) (64). Thirty-seven were observational studies, and four were interventional studies. The former were generally cross-sectional studies aimed at describing the features of gait and dynamic balance assessment of the included sample. The latter mainly evaluated treatments’ effectiveness on patients’ functional ability. Fourteen studies focused on the Instrumented “Timed Up & Go” test (ITUG), while 27 focused on the Instrumented walk (IWALK).

Overall, 395 patients, aged 66 ± 5.9, were assessed with a wearable sensor in baseline condition.

### 3.2 Quality assessment

**Figure 2:**
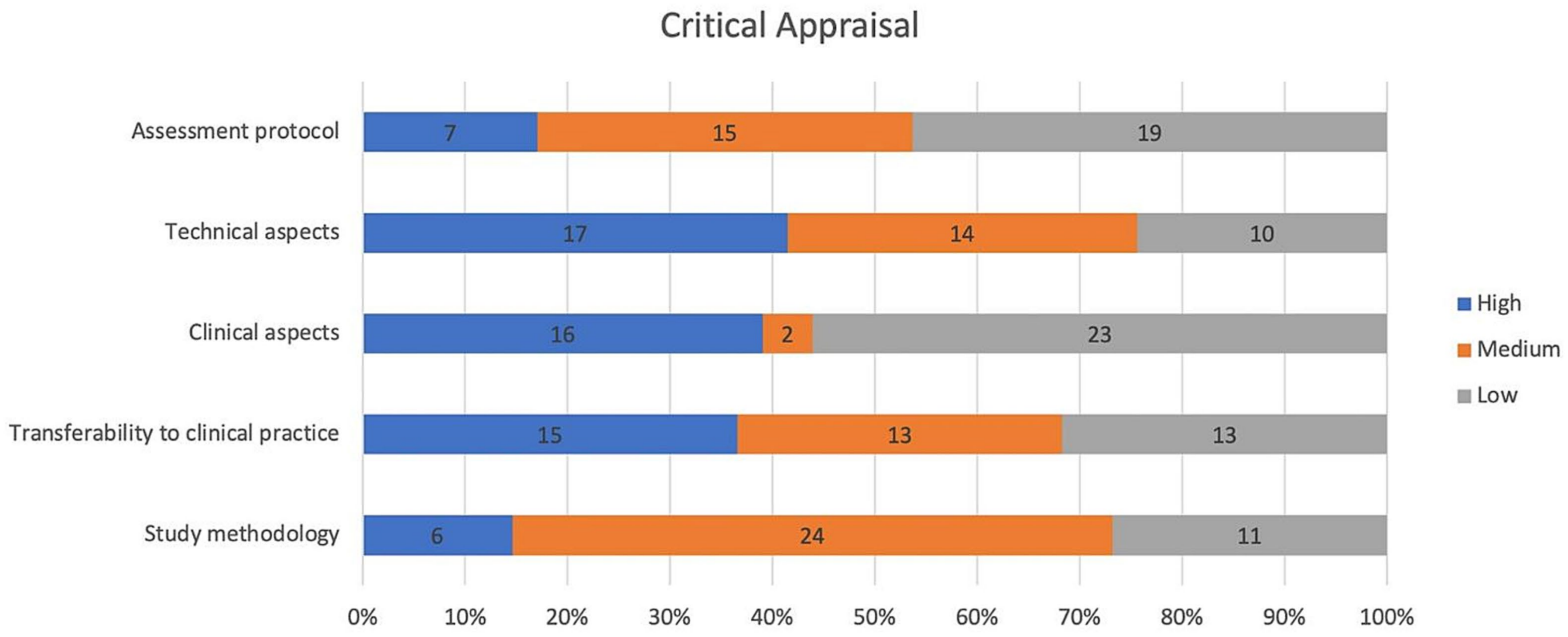
Quality assessment.

Of the 41 studies, five (12%) were rated as high-quality (60, 66, 78, 83, 90), 33 (80%) as medium-quality (23, 25–27, 30, 41, 61–65, 67–70, 72, 73, 75–77, 80–82, 84–89, 91, 92), and three (8%) as low-quality (34, 71, 93). The category “technical aspects” received high-quality ratings in 17 studies (see Figure 2). The “study methodology” category was predominantly rated as medium-quality. The lowest scores were observed in the “clinical aspects” and “assessment protocol” categories.

Table 3 presents the results for each item across the five domains, reporting the average rating scores (see Supplementary material 2, 3 for details of individual studies). As described in the Methods section, the total score for each question was calculated as a percentage, excluding items rated as “NA” to avoid penalizing studies for non-assessable questions. As a result, some ratios have denominators smaller than the total number of included studies.

**Table 3:** Results of the critical appraisal for each item.

Assessed domain	Results
Study methodology	
Q1 – Study aims	32/41 (78%) studies clearly stated the aim at the end of the introduction
Q3 – Study design	8/41 (20%) studies explicitly specified the study design
Q5 – Sample Characteristics	13/41 (32%) studies had an appropriate sample, while it was partially adequate in 25/41 (61%) studies.
Q6 – Sample size	6/41 studies (15%) only calculated the sample size *a priori* or performed a post-hoc power analysis. Of these: 4 were interventional studies. 2 were observational studies.
Q17 – Limitations	22/41 studies (54%) appropriately discussed study limitations, while 13/41 (32%) studies barely mentioned them. 6/41 (15%) studies completely omitted to report study limitations.
Q18 – Conclusions	36/41 (88%) authors wrote their conclusions consistently with the aims
Clinical aspects	
Q4 – Sample Description	18/41 (44%) of the studies accurately listed the eligibility criteria
Q7 – Intervention description	2/4 (50%) of the interventional studies described the intervention protocol in detail, allowing reproducibility
Assessment protocol	
Q9 – Protocol Description	16/41 (39%) studies accurately described the acquisition protocol, while 25/41 (61%) described it sufficiently. In the ITUG studies, Pathway length was: 3 m in 10/14 (72%) studies 7 m in 2/14 (14%) studies Not reported in 2/14 (14%) studies The number of trials acquired for the assessment was: 2 trials in 6/14 (43%) studies 3 trials in 8/14 (57%) studies The sensor was placed: between L3 and L5 vertebrae in 1 study on L4–L5 inter-vertebral space in 3/14 (22%) studies on L5 vertebra in 1 study on S1 vertebrae in 2/14 (14%) on lower back in 7/14 (50%) In the IWALK studies, Pathway length was: 7 m in 1 study 10 m in 11/27 (40%) studies 12 m in 1 study 14 m in 1 study 16 m in 1 study 18 m in 2/27 (7%) studies 20 m in 3/27 (10%) studies 30 m in 4/27 (15%) studies 36 m in 1 study 50 m in 1study Not reported in 1 study The number of trials acquired for the assessment was: 1 trial in 10/27 (36%) studies 2 trials in 5/27 (19%) studies 3 trials in 5/27 (19%) studies 4 trials in 1 study 5 trials in 1 study Not reported in 5/27 (18%) studies The sensor was placed: on L2 vertebra in 1 study on L3 vertebra in 1 study on L4 vertebra on 3/27 (11%) studies on L4–L5 inter-vertebral space in 2/27 (7%) studies on L5 vertebra in 11/27 (40%) studies on lower back in 5/27 (19%) studies on waistline in 4/27 (15%) studies
Q10 – Task Condition	6/41 (15%) studies appropriately detailed the task conditions, while 32/41 (78%) authors provided only limited information.
Q11 – Drug and Stimulation conditions	14/41 (34%) studies adequately specified the drug or stimulation conditions during the assessment (e.g., reporting the mean time since medication intake or the duration of the washout period). Similarly, one study involving DBS clearly described the stimulation parameters in the Methods section, including the frequency, stimulus voltage, and the elapsed time between DBS onset and the assessment.
Technical aspects	
Q8 – Data acquisition	5/41 (12%) studies correctly described both the sampling frequency and the filtering process, only 1 study described sampling frequency, filtering process and sensitivity. The rest on the studies (30/41 (73%)) reported only the sampling frequency, with no specification about the filters applied. Devices from 18 different producers were used, including: three-axial accelerometers only in 13/41 (32%) studies three-axial accelerometers and three-axial gyroscopes in 5/41 (12%) studies three-axial accelerometers, three-axial gyroscopes and magnetometer in 16/41 (39%) studies no information reported in 2/41 (5%) studies
Q14 – Parameter Computation	15/41 (37%) studies provided a thorough description of the computation process, including formulas, references, or clear definitions of the parameters. However, 16 of 41 studies (39%) did not provide any reference for parameter calculation, and 10 of 41 studies (24%) only partially described the formulas and computation methods
Q15 – Units of measurement	38/41 (93%) studies uniformly made explicit the units of measurements, while three studies (7%) reported only for a few parameters or are not consistent across the manuscript
Q16 – Parameter reliability	30/41 (73%) studies reported values consistent with current literature, while 11/41 (27%) studies presented values even greater than three median absolute deviations away from the median for at least one parameter
Transferability to clinical practice	
Q2 – Aims and clinical applications	22/41 (54%) studies were almost always explicitly in stating objectives related to clinical practice
Q12 – Rationale behind the protocol	18/41 (44%) studies never made explicit the rationale behind the protocol selection; in 20/41 studies (49%) the rationale could be derived from the text despite not being made explicit.
Q13 – Rationale behind the parameters	17/41 (42%) studies clearly stated the rationale behind the choice of the parameters, while it was completely omitted in 4 (10%) studies
Q19 – Discussion of clinical utility	16/41 (39%) studies made explicit the clinical utility, while for 20/41 could be inferred from the discussions

### 3.3 Differences in critical appraisal scores due to the journal subject area

Of the included studies, 20/41 were classified as strictly clinical (23, 26, 27, 30, 41, 61, 67, 68, 70, 73, 76, 81, 83, 84, 89, 90), while 21/41 were mixed studies (60, 62–64, 66, 69, 71, 72, 75, 77–80, 82, 85–87, 91, 92). Among the clinical studies, two (7%) were rated as high-quality (83, 90), 18 (93%) as medium-quality (23, 26, 27, 30, 41, 61, 67, 68, 70, 73, 76, 81, 84, 89), and none as low-quality. For the 21 mixed studies, three (14%) were rated high-quality (60, 66, 78), 15 (72%) medium-quality (25, 62, 63, 65, 72, 74, 75, 77, 79, 80, 82, 85–87, 91), and three (14%) low-quality (34, 71, 93).

**Figure 3:**
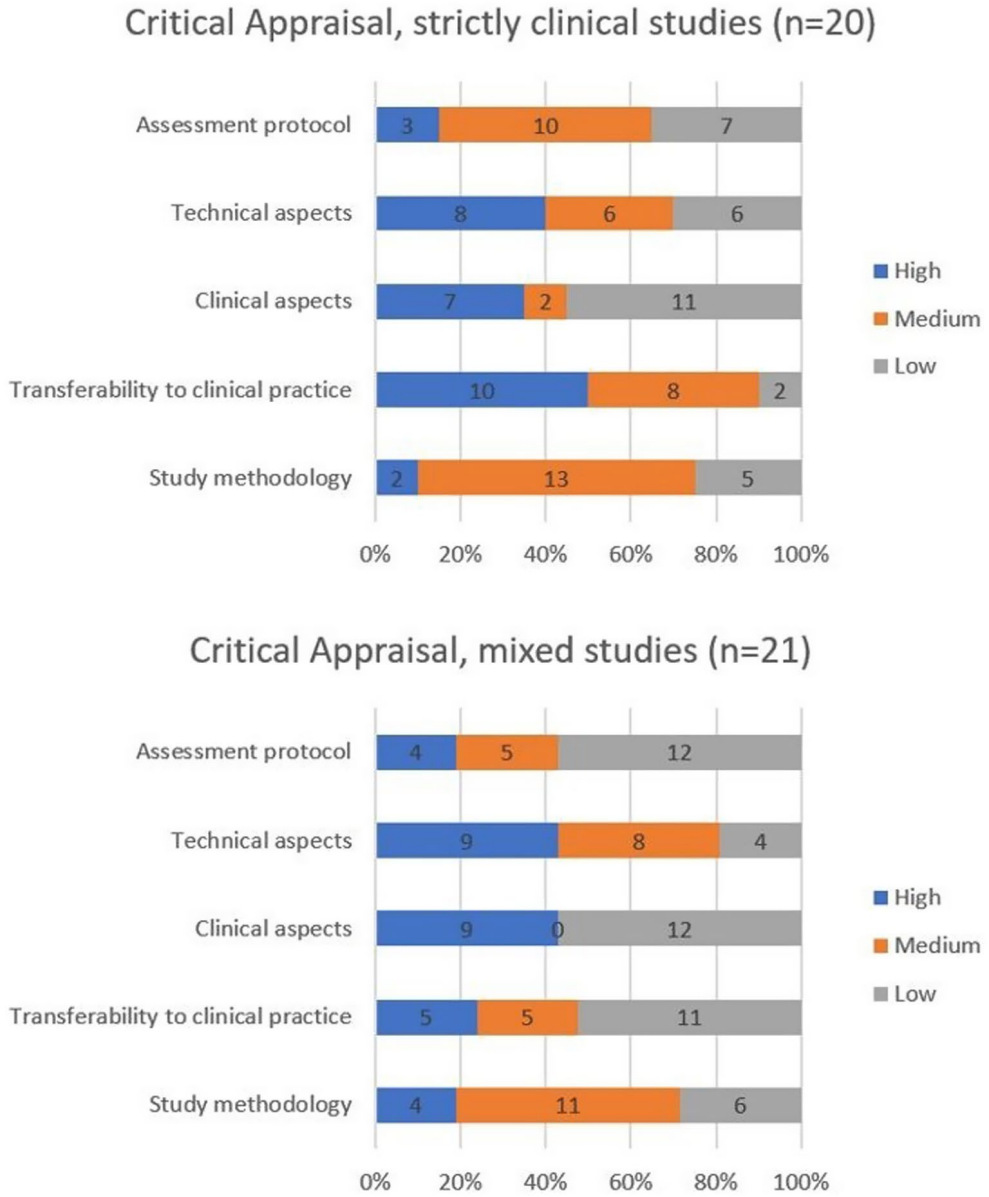
Differences in critical appraisal scores due to the journal subject area.

For clinical studies, the domain with the highest scores was “transferability to clinical practice” (see Figure 3A). In contrast, the “clinical aspects” category received the lowest scores, primarily because only half of the studies (27, 41, 67–70, 73, 83, 84) adequately described the sample characteristics (Q4), as discussed later. Although two clinical studies were classified as high-quality overall (83, 90), no study received high-quality ratings across all five domains.

For mixed studies, the highest scores were observed in the “technical aspects” and “clinical aspects” domains (see Figure 3B), while the lowest scores were found in the “clinical aspects” and “assessment protocol” categories. Four mixed studies received a high-quality global evaluation (60, 66, 78, 79), and one study achieved high-quality ratings for all five domains (78).

Notably, more than half of the studies received medium-quality scores for the “study methodology” domain (25, 26, 30, 63, 65, 72, 77, 80, 82, 85, 87).

### 3.4 Differences in critical appraisal scores based on the examined functional task

Of the included studies, 14/41 were assessing ITUG (23, 25–27, 34, 67, 69, 75–78, 85, 90, 92), and 27/41 were assessing IWALK (26, 27, 30, 41, 60–66, 68, 70–74, 79–83, 86–89, 93).

Of the 14 studies assessing ITUG, two (14%) were rated high-quality (78, 90), 11 (79%) medium-quality (23, 25–27, 67, 69, 75–77, 85, 92), and one (7%) low-quality (34). Among the 27 studies assessing IWALK, four (11%) were rated high-quality (60, 66, 79, 83), 22 (81%) medium-quality (30, 41, 61–65, 68, 70, 72–74, 80–82, 84, 86–89, 91, 93), and one (8%) low-quality (71).

**Figure 4:**
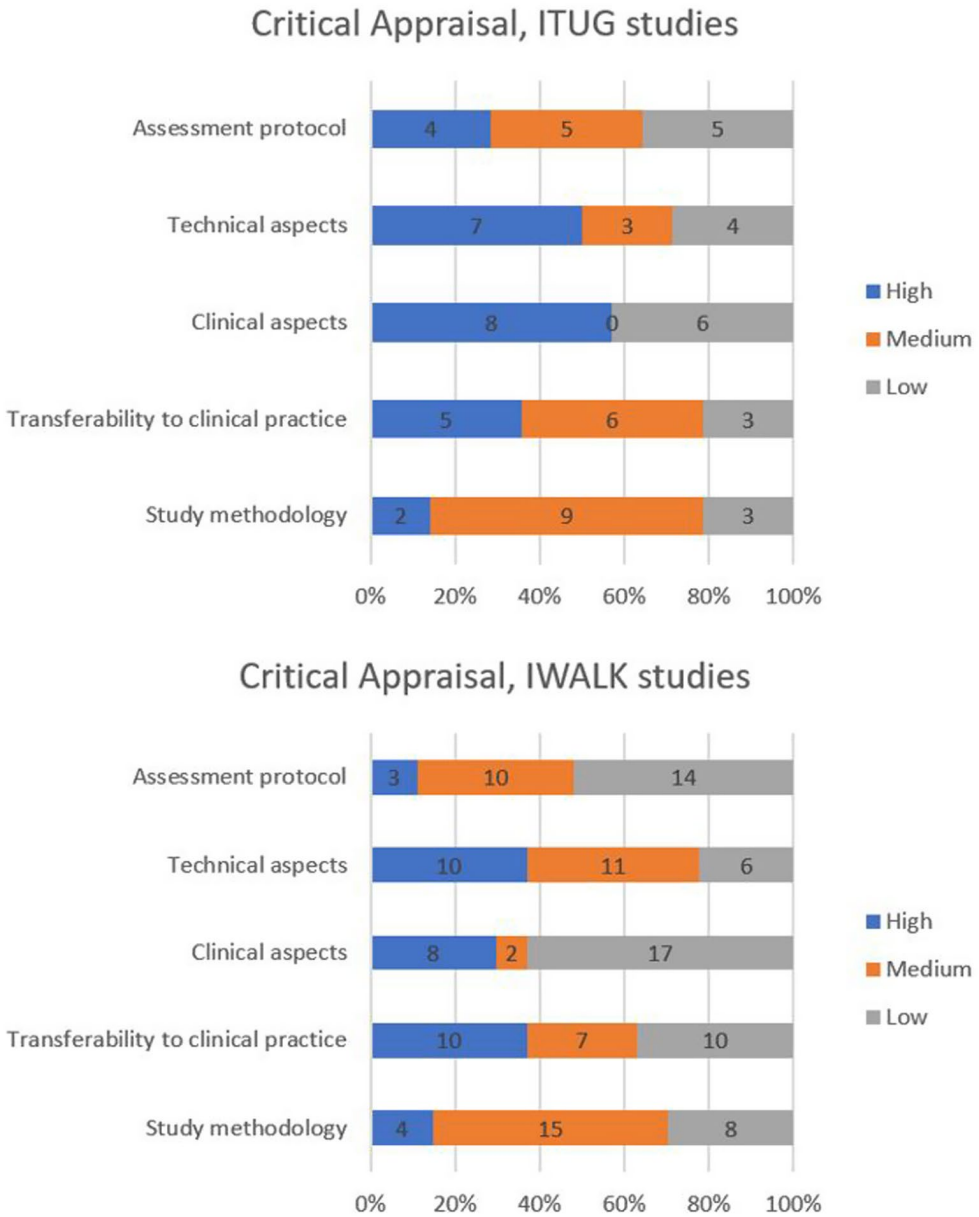
Differences in critical appraisal scores based on the examined functional task.

Regarding ITUG studies, the domain with the highest scores was “clinical aspects” (see Figure 4A). One study received high-quality ratings for all five domains (78).

Regarding IWALK studies, the domain with the highest score was “technical aspects” and trasferibility to clinical practice (see Figure 4B). The category with the lowest scores was “clinical aspects.” Among the mixed studies, four received a high-quality global evaluation (60, 66, 79, 83), but no study achieved high-quality ratings across all five domains. Notably, 17 of 27 studies were rated as low-quality in the “clinical aspects” domain (26, 30, 61–64, 66, 71, 72, 74, 81, 82, 86–89, 93).

### 3.5 Parameters extracted for data interpretation

Across the included studies, different outcome measures were derived from the inertial data to quantify performance during the functional tasks. Fourteen studies analyzed the instrumented Timed Up & Go (iTUG). Within these studies, the most frequently reported outcomes described the temporal structure of the task, particularly the total duration of the test and the duration of specific sub-phases (sit-to-stand, turning phase, and stand-to-sit). Although these measures were generally consistent across studies, some one reports (34) showed values that markedly deviated from the expected range. In this case, the mean task duration was about 24 s (compared to a median of 10.6 s among studies) and resulted from a sample of five patients with HY comprised between 2 and 3.

The remaining 27 studies examined instrumented straight-line walking (iWALK). In this group, the most frequently extracted outcomes described the spatiotemporal features of gait, particularly walking speed, step length, cadence, and step duration. Also in this case, cross-study comparison revealed the presence of outliers, especially for cadence, step time and step duration (26, 83, 88), for which the values referring to the stride, rather than to the step, were reported, thus resulting in doubled values. Similarly, the total double support time (i.e., left plus right) was reported by one author (64). Finally, stride length was exceedingly high (1.7 m) in the paper by Liguori et al. (80). These discrepancies may reflect differences in processing methods, task instructions, or sample characteristics, and highlight the need for more standardized reporting of parameter computation.

## 4 Discussion

This systematic review was conducted to evaluate the use of single-point mIMUs for gait and dynamic balance assessment in pwPD and atypical parkinsonism, focusing on the level of detail reported and its potential impact on clinical transferability. Based on the domains assessed by our appraisal tool, the articles classified as low quality in this review achieved high scores in the assessment of technical aspects only. Articles resulted of medium-quality were characterized by medium-to-high scores in all the assessed domains. The most critical issues identified in these studies concerned, on the one hand, the sample—particularly the accurate reporting of its characteristics and the *a priori* design of the sample size—and, on the other hand, the study design itself, especially due to the lack of a clear rationale underlying the selection of the experimental protocol and the instrumental indices selected. These issues were not present in the articles classified as high-quality studies.

The following sections examine these three issues in detail, their implications for clinical transferability, and potential strategies to address them.

### 4.1 The rationale supporting the instrumented gait and dynamic balance assessment of pwPD in the literature

Although rarely highlighted as a primary focus in the included studies, three main clinical applications of wearable sensors for gait and dynamic balance assessment in pwPD emerge from this review: (1) evaluating the effectiveness of rehabilitation treatments (four clinical trials); (2) monitoring disease progression; and (3) distinguishing subgroups of patients (37 observational studies).

Nineteen studies have explicitly discussed how the assessment protocol and investigated parameters can be used in clinical routine (23, 25–27, 41, 68, 69, 73, 78, 79, 82–84, 86–88, 90–92). Interestingly, the absence of a clear rationale for protocol and parameter selection is particularly evident in studies published in purely clinical journals (see Figure 3A), which might be expected to provide more detailed methodological justification. This lack of clarity makes it challenging for readers to translate the findings into routine clinical practice.

Several factors may account for this limitation. It is plausible that the authors assumed the rationale for their methodological choices was self-evident, given their routine use of such tools and parameters. Moreover, their primary focus may have been on the research-oriented aspects of their studies, resulting in limited attention to the clinical rationale and relevance of their protocols and findings. In addition, some journals do not explicitly require authors to address these dimensions, which may have further contributed to their omission.

With regard to the selection of functional parameters in the included studies, it is likely that these were influenced—or restricted—by the software capabilities of the commercial devices employed. Such systems typically offer only standard, widely used outcome measures, such as gait speed or total task duration. While these parameters are common, they may not be the most informative or clinically meaningful in the assessment of pwPD (52, 94).

It is well established that the integration of research evidence into clinical practice may take up to two decades (95). To narrow this gap, scientific literature should place greater emphasis on the clinical relevance and transferability of research findings. Increasingly, journals require a concise section on clinical implications as part of manuscript submissions. In alignment with this trend, future studies should explicitly address the added value of functional tests beyond standard clinical assessments. Specifically, they should articulate the clinical importance of such evaluations in pwPD and clarify how the selected assessment protocols—both in terms of tasks and parameters—may contribute to differential diagnosis, early symptom detection, treatment effect evaluation, or longitudinal monitoring of patient status.

### 4.2 Characteristics of pwPD assessed by wearable sensors in the literature

This systematic review included both pwPD and individuals with atypical parkinsonism. To our knowledge, this represents a novel contribution to the literature, as it is the only review to consider both populations. Although less common, conditions such as PSP can present with clinical symptoms at onset that resemble PD, highlighting the importance of investigating and differentiating these populations.

#### 4.2.1 The choice and description of the sample

Forty-four percent of the included studies reported complete details about the sample, which are essential for ensuring the external validity of the results. Providing accurate sample characteristics enables readers to determine whether the findings are comparable to their own patient populations. High-quality studies were similarly distributed, with 35% published in clinical journals and 43% in mixed journals. This shows an improvement with respect to another paper published by our group (47), in which the results were likely due to a greater sensitivity of clinical teams in reporting aspects that characterize the sample, compared to engineering groups.

For pwPD, many studies reported diagnostic criteria and characterized patients through the Unified Parkinson’s Disease Rating Scale (UPDRS) (96) and the Hoehn and Yahr staging (H&Y) scales (97), as well as additional functional tests. For patients with atypical parkinsonism, a variety of specific scales can be used, depending on the disease, including the Unified Multiple System Atrophy Rating Scale, Progressive Supranuclear Palsy Rating Scale, and Scale for the Assessment and Rating of Ataxia (98, 99). In progressive diseases such as PD, it is important to report the time since onset of the first symptoms or diagnosis, as well as participants’ age and gender. In this review, a sample was considered “adequate” when it included patients with homogeneous characteristics for intervention or descriptive studies, and heterogeneous characteristics for correlation studies (see Q5). Among the included studies, 32% defined inclusion criteria that were consistent with the study’s aims. Ensuring sample adequacy enables authors to identify specific findings for well-defined subgroups in terms of disease severity, thereby supporting the translation of results into clinical practice. Additionally, authors should clarify whether the study was conducted solely for research purposes or integrated into standard clinical care.

When conducting clinical studies, the intervention should also be clearly described. In this review, 50% of studies provided detailed protocols, including the type of intervention, frequency, and dosage, allowing for reproducibility (see Q7). The TIDieR checklist was specifically developed in 2014 to improve the reporting quality for healthcare interventions and may further support authors during the writing of their Methods section (100).

#### 4.2.2 The methodology used

The methodology domain contributed significantly to the overall lower quality of the included studies. Across all studies, only 15% met the criteria for high methodological quality, and this proportion decreased to 10% when considering only studies published in clinical journals.

The main methodological limitation found in the included studies is the rare *a-priori* sample size calculation (see Q6). Only 4 out of 41 studies (10%) reported having conducted *a priori* sample size estimations, with one additional study reporting a *post hoc* power analysis. Focusing specifically on interventional studies, only 50% employed an adequately calculated sample size. This constitutes a major methodological flaw, as the absence of appropriate sample size estimation increases the risk of type II errors—i.e., failing to detect a true difference in instrumental outcomes between experimental and control groups, when it actually exists. Consequently, the lack of such calculations undermines both the generalizability and the clinical applicability of the study findings. Furthermore, it hinders the observation of potential differences between groups (in both observational and clinical studies), thereby impacting clinical decision-making (101).

When evaluating methodology, it is also important for studies to clearly acknowledge their own limitations, which helps readers avoid overestimating the results and remain aware of potential challenges when attempting to replicate the work in clinical practice. In this review, 22 of 41 studies comprehensively reported their limitations (see Q17), but authors rarely cited the reporting guidelines followed. Since the late 1990s, several reporting guidelines have been published, including CONSORT for experimental studies (1996) (102) and STROBE for observational studies (2007) (103). Increasingly, journals require these guidelines to be completed during submission, highlighting their usefulness in ensuring methodological rigor and the transparent reporting of all necessary information.

### 4.3 Issues in the reporting of protocol details and technical information in the literature

A difference in quality was observed across domains: “assessment protocol” included only 7 high-quality studies (17%), while “technical aspects” included 17 (42%). Besides that, many studies obtained fair scores in the critical appraisal by providing the necessary information. However, several technical and assessment-related aspects still warrant further attention.

#### 4.3.1 Protocol description

Among the 41 studies included in this review, only 16 (39%) provided a detailed and accurate description of the acquisition protocol, whereas the remaining 25 studies (61%) presented the protocol with sufficient, albeit not comprehensive, detail. When focusing on studies utilizing the ITUG test (*n* = 14), a considerable degree of homogeneity was observed in the walkway length, with the majority of studies (10/14, 72%) employing a standardized 3-meter pathway.

In contrast, studies utilizing the IWALK (*n* = 27) demonstrated greater methodological heterogeneity. The most frequently reported walkway length was 10 meters, employed in 11 studies (40%).

Overall, these findings highlight a lack of methodological consistency in protocol descriptions, particularly within IWALK studies. While ITUG protocols showed greater uniformity, especially concerning walkway length and trial repetition, a substantial variability in sensor placement was displayed. The placement of the IMU near the lower lumbar spine, often close to L4, is commonly adopted due to its anatomical plausibility and alignment with the body’s estimated CoM (104). This location is chosen because the fascia over the spinous processes in this region is relatively tightly bound to the underlying bone, which may help reduce soft tissue artifacts and ensure that skin movement approximates vertebral motion (105). However, current literature does not provide direct comparative studies evaluating metric performance across different lumbar levels, and the propagation of measurement errors to derived gait parameters has not been systematically investigated. Additionally, accurately distinguishing between adjacent vertebral levels (e.g., L4 vs. L5) can be challenging in clinical practice due to anatomical variability and sensor size. Therefore, lower lumbar placement should be considered a commonly used and anatomically plausible option rather than a standardized or superior reference location, highlighting the need for future comparative investigations.

##### 4.3.1.1 Task instructions

Task instructions are another critical factor for the clinical transferability of gait and dynamic balance assessment. Among the 41 studies included in this review, only six studies (15%) provided a comprehensive and appropriate description of the task conditions under which data acquisition was performed. The majority of studies (32/41, 78%) offered only limited information concerning task execution, which poses challenges for reproducibility and interpretation of results. Specifically, 23 studies (56%) clearly reported the verbal instructions given to participants prior to or during task execution. However, other key contextual variables were frequently underreported. Only six studies (15%) explicitly stated that participants performed the tasks barefoot, while other two studies (5%) reported the use of footwear. This lack of information regarding footwear is particularly relevant given its potential influence on gait parameters and sensor-derived measurements (106, 107).

In addition to baseline gait conditions, a subset of studies investigated alternative task conditions aimed at increasing the ecological validity and cognitive or motor complexity of the walking assessment. Two studies assessed performance under a fast-paced walking condition, while six studies (15%) implemented a dual-task paradigm. In dual-task protocols, participants were typically instructed to perform a simultaneous cognitive or motor task while walking. These tasks included counting backwards, generating as many words as possible beginning with a given letter or belonging to a specific semantic category, engaging in general verbal fluency tasks, or executing an additional motor activity. Despite the relevance of these enriched paradigms for evaluating real-world functional mobility, their inconsistent implementation and limited reporting highlight a significant methodological gap. Overall, the lack of standardized and thoroughly described task conditions across studies represents a critical limitation, potentially influencing gait performance and undermining comparability across studies. Future research should aim to improve transparency in the reporting of task parameters, including verbal instructions, footwear conditions, and secondary tasks, to ensure methodological rigor and facilitate meta-analytical synthesis.

Finally, because pwPD are almost always on a pharmacological regimen, it is important to specify the exact phase (ON/OFF) during which the assessment was conducted; simply stating whether the patient was in the ON or OFF phase is not sufficient. Only 14 (34%) adequately reported the pharmacological conditions under which assessments were performed. Specifically, these studies provided essential methodological details such as the mean time elapsed since medication intake or the duration of the wash-out period prior to assessment. This level of precision is crucial when evaluating motor performance in populations affected by neurological conditions, particularly PD, where motor fluctuations are closely tied to dopaminergic medication cycles. If in OFF, the patient must perform a wash-out of at least 12 h; if in ON, the peak of maximum action occurs about 1–2 h after the last intake (108).

Two studies included participants undergoing deep brain stimulation (DBS) and provided sufficient methodological detail regarding stimulation parameters. These studies clearly specified key aspects of the DBS configuration, including the stimulation frequency, stimulus voltage, and the time interval between DBS activation and the commencement of the assessment. Such information is essential to interpret the observed motor performance accurately, as the neuromodulatory effects of DBS may vary depending on both the stimulation parameters and the duration of their application.

#### 4.3.2 Technical aspects

Details of data analysis should always be reported in manuscripts due to their impact on the results and to ensure reproducibility.

In particular, procedures such as mean value removal, data filtering, parameter calculation, and subsequent synthesis methods (e.g., computing the mean or median across trials, excluding the first and last seconds of each trial) should be clearly described. Any use of filters or analytical techniques that deviate from standard practice should be explicitly justified.

Recent evidence highlights the critical importance of selecting and reporting appropriate filter settings when processing IMU data, particularly in high-intensity motor tasks. Based on controlled perturbation experiments involving trunk kinematics, Miller et al. (109) recommend that filter cut-off frequencies should not be determined solely by the type of activity or the body segment under investigation, but rather by the specific characteristics of the recorded signal—most notably, the magnitude of segmental accelerations.

Both studies published in clinical and mixed journals (i.e., those with engineering contributions) addressed technical aspects similarly, with 40% of mixed-journal studies and 43% of clinical-journal studies rated as high-quality. Overall, however, a lack of standardization was observed in data acquisition, filtering methods, and parameter computation when reported. For example, the low-pass filtering frequency—a factor with a significant impact on computed parameters—was rarely specified, appearing in only five studies (12%). This lack of reporting prevents meaningful comparisons between studies and limits the possibility of synthesizing result, although significant progress has been made in the standardization of mIMU signal processing. Details on pre-processing steps—including calibration, sensor-fusion–based orientation estimation, gravity removal, and band-pass filtering—are available in the methodological framework and technical validation results of the Mobilise-D project.^2^ This framework provides practical guidance to support standardization in signal processing across different functional assessments.

##### 4.3.2.1 Instrumental set-up

Technical specifications regarding mIMUs were inconsistently reported across the 41 studies reviewed. Only a small proportion of studies (5/41, 12%) provided a complete and appropriate description of both the sampling frequency and the filtering processes applied to the raw data. Notably, just one study (2%) reported all three critical technical parameters: sampling frequency, filter characteristics, and sensor sensitivity. In contrast, the majority of studies (30/41, 73%) reported only the sampling frequency, omitting essential information about signal preprocessing, such as the type of filters used, their cutoff frequencies, and the rationale behind their application. The lack of transparency in describing filtering procedures limits the reproducibility of findings and hinders the accurate interpretation of sensor-derived measures, particularly those related to dynamic gait features, which are sensitive to signal processing methods.

Furthermore, a wide range of hardware configurations was observed across the included studies, with devices from 18 different manufacturers being employed. In terms of sensor composition, 13 studies (32%) utilized devices equipped exclusively with tri-axial accelerometers, while five studies (12%) combined tri-axial accelerometers with tri-axial gyroscopes. A more comprehensive sensor suite, including tri-axial accelerometers, gyroscopes, and magnetometers, was used in 16 studies (39%). However, two studies (5%) failed to report any information regarding the technical specifications or sensor modalities of the IMU employed, further highlighting the variability and, at times, inadequacy of methodological reporting in this field.

##### 4.3.2.2 Computation of the functional parameters

Among the included studies, 15 (37%) provided a thorough and transparent description of the computational process used to derive biomechanical parameters from inertial sensor data. These studies clearly reported formulas, defined all variables, and cited relevant methodological references, ensuring clarity and full reproducibility. In contrast, 16 studies (39%) did not provide any methodological references or explanations regarding how parameters were calculated, while an additional 10 studies (24%) offered only partial details—for example, mentioning formulas without defining the variables involved or omitting specific steps of the computational pipeline. Regarding reporting standards, most studies (40/41) consistently stated the units of measurement, thereby improving the interpretability of results; however, one study reported units inconsistently for some outcome measures (75). For adimensional acceleration-derived metrics, no measurement units are applicable; therefore, these metrics were not considered among the studies with missing unit reporting.

With respect to parameter validity, 31 studies (76%) reported values that were homogeneous among the selected papers, suggesting sound methodological procedures. Conversely, 10 studies (24%) presented values that were statistical outliers—defined as greater than three median absolute deviations from the median—for at least one parameter. These inconsistencies raise concerns about possible methodological errors, atypical participant characteristics, or non-standardized data processing. In most cases, discrepancies were attributable to a different definition assigned to the same variable, such as cadence being reported in strides/min rather than in steps/min. Finally, the use of angular velocities and accelerations should be more widely encouraged when working with inertial sensors. Recent studies have shown that peak angular velocity during turning, as well as features derived from trunk accelerations during the iTUG, can discriminate between patient subgroups or predict clinical outcomes (110). Likewise, during walking, parameters related to gait regularity and symmetry extracted from trunk accelerations—particularly when assessed over longer distances (e.g., back-and-forth corridor walking)—have proven effective in characterizing patients’ motor status (60, 73). Together, these findings suggest that metrics beyond conventional spatiotemporal parameters may better capture subtle gait deficits in pwPD and should be more consistently considered in future studies.

##### 4.3.2.3 Main barriers to clinical applicability

Clinical translation of wearable sensor technologies in Parkinson’s disease is currently hindered by substantial methodological heterogeneity, particularly variability in sensor placement and the absence of standardized acquisition protocols, including inconsistent walkway lengths and task instructions. In addition, incomplete reporting of technical parameters (e.g., filtering methods and computational formulas) and insufficient clinical characterization—such as unclear pharmacological (ON/OFF) or stimulation states—limit reproducibility and cross-study comparability. Detailed clinical characterization of patient samples should also be ensured to enhance interpretability and external validity. Expanding research to include patients with atypical parkinsonism would further broaden the applicability of wearable technologies beyond idiopathic Parkinson’s disease.

Ultimately, interdisciplinary collaboration among clinicians, engineers, and data scientists will be essential to bridge the gap between technological innovation and clinical implementation, accelerating the adoption of wearable sensor systems in everyday neurological practice.

### 4.4 Limitations

This review has some limitations that need to be considered: although we included studies on both PD and atypical parkinsonism, UPDRS scores were inconsistently reported across studies, preventing stratification by disease severity. This is important because PD is a highly heterogeneous condition, and differences in severity could influence gait and balance outcomes, potentially affecting the generalizability of our findings.

In this review, we included only studies that employed single-point mIMUs for gait and dynamic balance assessment. Although multi-point IMU systems have been validated against gold-standard methods (111, 112) and are increasingly used in clinical settings to analyze gait parameters (113–115), they were excluded from our analysis. These systems can provide additional valuable insights for clinical practice; however, their broader implementation is limited by challenges related to sensor placement complexity, user intrusiveness, and consistency across sessions (116). While single-point mIMUs may not match the accuracy and reliability of multi-point configurations (117), they have nonetheless demonstrated substantial potential for gait and dynamic balance assessment in both research and clinical contexts.

The search was conducted across four databases and supplemented by manual screening of reference lists; however, some relevant studies not indexed under the chosen keywords may have been missed. Additionally, the critical appraisal tool used to assess the included studies was developed *ad hoc* by the authors.

Although we relied on similar examples to conceive the items (47, 48) and develop the scoring system (48), followed an iterative process, and consulted with experts for its design, it is possible that some important aspects were not considered. The tool should therefore be considered a study-specific instrument aimed at highlighting field-specific methodological gaps rather than a generalizable metric.

### 4.5 Suggestion for future studies

Future research should prioritize the development and adoption of standardized protocols for sensor placement, task execution, and data processing to improve cross-study comparability and clinical relevance. Building on the methodological gaps identified in our appraisal and in alignment with PRISMA principles and prior sensor-based reviews, we propose the following structured framework to foster standardization without over-prescription:

- Clinical characterization: Provide detailed reporting of UPDRS and Hoehn & Yahr staging, disease duration, pharmacological (ON/OFF) and stimulation status, and other relevant clinical features to enhance interpretability and reproducibility.
- Protocol standardization: Clearly describe task execution, including exact sensor placement (e.g., L5–S1), number of repetitions, walkway length, footwear conditions, and instructions reported verbatim.
- Technical transparency: Report sampling rate, filtering procedures, signal processing steps, and formulas or references for all computed parameters to ensure replicability.
- Rationale alignment: Explicitly link selected tasks and outcome parameters to the study aims (e.g., progression monitoring, fall risk assessment, treatment response), providing a clear justification for parameter selection.
- Transferability and methodological rigor: Include *a priori* sample size estimation, discuss study limitations transparently, and adhere to established reporting guidelines (e.g., STROBE or CONSORT) to strengthen robustness and facilitate clinical adoption.

Moreover, future studies should expand research to patients with atypical parkinsonism to broaden the applicability of wearable technologies beyond idiopathic Parkinson’s disease.

## 5 Conclusion

This systematic review evaluated the use of single-point mIMUs for gait and dynamic balance assessment in pwPD and individuals with atypical parkinsonisms, focusing on the methodological, clinical, technical, and translational aspects of the included studies. While wearable sensors represent a promising tool for improving the objectivity and ecological validity of gait and dynamic balance assessments, the overall quality of the literature remains moderate. Major limitations include insufficient methodological transparency, lack of standardized assessment protocols, heterogeneous reporting of clinical and instrumental parameters, and inadequate articulation of clinical rationale. These issues collectively hinder the reproducibility of findings and limit their applicability in routine clinical settings. Nevertheless, several high-quality studies have demonstrated the feasibility and potential clinical utility of single-sensor configurations, particularly when rigorous reporting standards are followed. This review provides a structured framework and practical recommendations to enhance future research, promoting greater methodological consistency and improving the transferability of sensor-based assessments into clinical practice. Widespread adoption of these tools will depend not only on technological accuracy, but also on the alignment of research methodologies with clinical needs and reporting transparency.

## Data Availability

The original contributions presented in the study are included in the article/Supplementary material, further inquiries can be directed to the corresponding author.

## Footnotes

1
https://www.scimagojr.com

2 See https://www.mobilise-d.eu/.
