# Supplementary material for: The use of single-point magneto-inertial measurement for gait and dynamic balance assessment of patients with Parkinson’s disease and Parkinsonisms: a systematic review with critical appraisal of clinical applications and quality of reporting
Source: Front Neurol. 2026 Mar 25;17:1758979. doi: 10.3389/fneur.2026.1758979 (PMC13056617; doi:10.3389/fneur.2026.1758979)
Supplement: Supplementary file 1 [file Table_1.DOCX]

| Database | Strategy |
| --- | --- |
| Medline (PUBMED) | (Parkinson* OR canvas OR “multisystem* atrophy” OR “progressive supranuclear palsy” OR corticobasal OR “GBA Parkinson” OR "Parkinson Disease"[Mesh] OR "Parkinsonian Disorders"[Mesh]) AND (Walk[Title/Abstract] OR Gait[Title/Abstract] OR "Instrument*"[Title/Abstract] OR "Timed Up and Go"[Title/Abstract] OR "Timed Up & Go"[Title/Abstract] OR TUG[Title/Abstract]) AND (“Wearable” [Title/Abstract] OR “Acceleration” [Title/Abstract] OR “Acceleration” [Mesh] OR “Accelerometer” [Title/Abstract] OR “Gyroscope” [Title/Abstract] OR “Inertial” [Title/Abstract] OR “Inertial Measurement Unit” [Title/Abstract] OR “IMU” [Title/Abstract]) |
| EMBASE | (Parkinson* OR canvas OR 'multisystem* atrophy' OR 'progressive supranuclear palsy' OR corticobasal OR 'GBA Parkinson' OR 'Parkinson Disease'/exp OR 'Parkinsonian Disorders'/exp)  AND (Walk:ti,ab OR Gait:ti,ab OR Instrument*:ti,ab OR 'Timed Up and Go':ti,ab OR 'Timed Up & Go':ti,ab OR TUG:ti,ab)  AND (Wearable:ti,ab OR Acceleration:ti,ab OR Acceleration/exp OR Accelerometer:ti,ab OR Gyroscope:ti,ab OR Inertial:ti,ab OR 'Inertial Measurement Unit':ti,ab OR IMU:ti,ab) |
| CINHAL | (Parkinson* OR canvas OR "multisystem* atrophy" OR "progressive supranuclear palsy" OR corticobasal OR "GBA Parkinson" OR (MH "Parkinson Disease+") OR (MH "Parkinsonian Disorders+"))  AND ((TI Walk OR AB Walk) OR (TI Gait OR AB Gait) OR (TI Instrument* OR AB Instrument*) OR (TI "Timed Up and Go" OR AB "Timed Up and Go") OR (TI "Timed Up & Go" OR AB "Timed Up & Go") OR (TI TUG OR AB TUG)) AND ((TI Wearable OR AB Wearable) OR (TI Acceleration OR AB Acceleration) OR (MH Acceleration+) OR (TI Accelerometer OR AB Accelerometer) OR (TI Gyroscope OR AB Gyroscope) OR (TI Inertial OR AB Inertial) OR (TI "Inertial Measurement Unit" OR AB "Inertial Measurement Unit") OR (TI IMU OR AB IMU)) |
| SCOPUS | (Parkinson* OR canvas OR "multisystem* atrophy" OR "progressive supranuclear palsy" OR corticobasal OR "GBA Parkinson" OR INDEXTERMS("Parkinson Disease") OR INDEXTERMS("Parkinsonian Disorders")) AND (TITLE-ABS(Walk) OR TITLE-ABS(Gait) OR TITLE-ABS(Instrument*) OR TITLE-ABS("Timed Up and Go") OR TITLE-ABS("Timed Up & Go") OR TITLE-ABS(TUG)) AND (TITLE-ABS(Wearable) OR TITLE-ABS(Acceleration) OR INDEXTERMS(Acceleration) OR TITLE-ABS(Accelerometer) OR TITLE-ABS(Gyroscope) OR TITLE-ABS(Inertial) OR TITLE-ABS("Inertial Measurement Unit") OR TITLE-ABS(IMU)) |
